# Rosmarinic acid treatment protects against lethal H1N1 virus-mediated inflammation and lung injury by promoting activation of the h-PGDS-PGD_2_-HO-1 signal axis

**DOI:** 10.1186/s13020-023-00847-0

**Published:** 2023-10-27

**Authors:** Beixian Zhou, Linxin Wang, Sushan Yang, Yueyun Liang, Yuehan Zhang, Xiping Pan, Jing Li

**Affiliations:** 1 The People’s Hospital of Gaozhou, Gaozhou, 525200 China; 2 State Key Laboratory of Respiratory Disease, National Clinical Research Center of Respiratory Disease, Guangzhou Institute of Respiratory Health, Institute of Chinese Integrative Medicine, Guangdong-Hongkong-Macao Joint Laboratory of Infectious Respiratory Disease, The First Affiliated Hospital of Guangzhou Medical University, Guangzhou Medical University, Guangzhou, Guangdong China; 3 Guangzhou Laboratory, Guangzhou, China

**Keywords:** H1N1 virus, Rosmarinic acid, Inflammation, Apoptosis, h-PGDS, PGD_2_, HO-1

## Abstract

**Background:**

Rosmarinic acid (RosA) is a natural phenolic compound that possesses a wide-range of pharmacological properties. However, the effects of RosA on influenza A virus-mediated acute lung injury remain unknown. In this study, we aimed to explore whether RosA could protect against H1N1 virus-mediated lung injury and elucidate the underlying mechanisms.

**Methods:**

Mice were intragastrically administered with RosA for 2 days before intranasal inoculation of the H1N1 virus (5LD_50_) for the establishment of an acute lung injury model. At day 7 post-infection (p.i.), gross anatomic lung pathology, lung histopathologic, and lung index (lung weight/body weight) were examined. Luminex assay, multiple immunofluorescence and flow cytometry were performed to detect the levels of pro-inflammatory cytokines and apoptosis, respectively. Western blotting and plasmid transfection with hematopoietic-type PGD_2_ synthase (h-PGDS) overexpression were conducted to elucidate the mechanisms.

**Results:**

RosA effectively attenuated H1N1 virus-triggered deterioration of gross anatomical morphology, worsened lung histopathology, and elevated lung index. Excessive pro-inflammatory reactions, aberrant alveolar epithelial cell apoptosis, and cytotoxic CD8^+^ T lung recruitment in the lung tissues induced by H1N1 virus infection were observed to be reduced by RosA treatment. In vitro experiments demonstrated that RosA treatment dose-dependently suppressed the increased levels of pro-inflammatory mediators and apoptosis through inhibition of nuclear factor kappa B (NF-κB) and P38 MAPK signaling pathways in H1N1 virus-infected A549 cells, which was accompanied by promoting activation of the h-PGDS-PGD_2_-HO-1 signal axis. Furthermore, we strikingly found that h-PGDS inhibition significantly abrogated the inhibitory effects of RosA on H1N1 virus-mediated activation of NF-κB and P38 MAPK signaling pathways, resulting in diminishing the suppressive effects on the increased levels of pro-inflammatory cytokines and chemokines as well as apoptosis. Finally, suppressing h-PGDS prominently abolished the protective effects of RosA on H1N1 virus-mediated severe pneumonia and lung injury.

**Conclusions:**

Taken together, our study demonstrates that RosA is a promising compound to alleviate H1N1 virus-induced severe lung injury through prompting the h-PGDS-PGD_2_-HO-1 signal axis.

## Introduction

The human respiratory system is always the first organ to be attacked by various types of respiratory viruses, such as influenza viruses [1]. Influenza-related acute contagious respiratory system illness caused by type A influenza strains can rapidly progress to life-threatening acute lung injury or even the severe form acute respiratory distress syndrome (ARDS) that ultimately leads to respiratory failure with high mortality rates [2]. The main histopathologic features of influenza-related acute lung injury are characterized by alveolar-capillary barrier disruption, uncontrolled pneumonia, interstitial pulmonary edema, apoptotic and necrotic alveolar epithelial cells [2–4]. Despite advances in antiviral agents and ventilator management, the high mortality rate of patients with influenza-related lung injury or ARDS even reaches a rate of 25.1–41% [5–7]. Accumulating evidence reveals that the imbalance of pro- and anti-inflammatory cytokine production as well as nonspecific apoptosis of uninfected alveolar epithelial cells are critical contributors that lead to the progression of critical influenza diseases. While the use of corticosteroids to reduce excessive inflammation was found to be associated with higher mortality in individuals with influenza illnesses [8]. Given the continuing threat of a future influenza pandemic, the development of specific and effective medicines for the treatment of severe influenza illness is of particular importance. It has been proposed that therapeutic modulation of the key host cellular molecular targets that could potentially attenuate the severe influenza pneumonia and the aberrant apoptosis may be a promising strategy for influenza illness treatment.

The hematopoietic-type PGD_2_ synthase (h-PGDS), a member of the sigma class glutathione-*S*-transferase family, is a rate-limiting enzyme that catalyzes the conversion of PGH_2_ to PGD_2_. h-PGDS is mainly expressed in hematopoietic lineage cells, such as macrophages, dendritic cells (DC), mast cells, and Th2 cells [9]. Recently, h-PGDS has received increasing attention due to its role in the resolution of inflammation and facilitating recovery from exogenous insults. Mice with h-PGDS deficiency had more severe methylated BSA-induced pro-inflammatory reactions than wild-type mice, whereas overexpression of h-PGDS was observed to lower these inflammatory responses [10]. It has been discovered that elevating PGD_2_ is involved in the anti-inflammatory activity of h-PGDS [11]. The physiological effects of PGD_2_ are transmitted by interaction with the two G-protein-coupled receptors, namely PGD_2_ receptor 1 (DP1) and 2 (DP2). Treatment with PGD_2_/DP1 pathway agonists significantly reduced bacteria-mediated production of a series of pro-inflammatory mediators, including IL-6, TNF-α, IL-1β and HMGB1 [12]. Moreover, the h-PGDS-PGD_2_-DP signal axis in nonhematopoietic alveolar and endothelial cells orchestrates endothelial barrier formation and inflammation resolution, thus protecting against LPS-mediated acute lung injury [13]. Of particular interest, PGD_2_ could be further nonenzymatically metabolized to PGJ_2_, Δ^12^-PGJ_2_ and 15d-PGJ_2_, which have been reported to possess binding affinity for PPAR-γ and activate PPAR-γ [14]. And activation of PPAR-γ by agonists is well-known for its capacity to suppress excess inflammation and alleviate lung injury in various viral infection scenarios, including influenza virus infection [15, 16]. However, whether the activation of the h-PGDS-PGD_2_-DP signal pathway in response to influenza virus infection plays a protective role has not been defined in previous studies. Based on the above-mentioned evidence that activation of the h-PGDS-PGD_2_-DP signal axis could be beneficial for the treatment of inflammation disorders, we hypothesized that modulation of h-PGDS-PGD_2_ activation by active compounds may be a novel strategy for alleviating influenza virus-triggered severe pneumonia and lung injury.

Many dietary plants, including fruits, vegetables, and cereals, contain naturally occurring bioactive phenolic chemicals that have been shown to have potential health-promoting benefits in the chemoprevention of various diseases [17]. Accumulating research has demonstrated that the potential health benefits of phenolic compounds have been ascribed to their diverse biological properties, for instance, anti-inflammatory [18], anti-apoptosis [19], antioxidant [18], anti-tumor [20], and anti-virus [21]. RosA (α-*O*-caffeoyl-3,4-dihydroxyphenyl lactic acid), a phenolic carboxylic acid, is abundant in several species of the Lamiaceae family (e.g., *Rosmarinus officinalis *L., *Ocimum basilicum *L., *Hyssopus officinalis *L.) [22]. Evidence from in vitro and in vivo studies has reported that treatment with RosA could provide therapeutic effects on a wide range of inflammatory diseases [23], including asthma, acute pancreatitis, allergic rhinitis, colitis and arthritis. It has been found that the broad-spectrum immunoregulation activities of RosA are due to its wide range of molecular targets, including NF-κB, MAPKs and P-STAT3 [24, 25]. Given that activation of the h-PGDS-PGD_2_-DP signal axis could be beneficial for inflammation disorder, it is yet unknown, though, if RosA treatment could stimulate the h-PGDS-PGD_2_ signaling pathway activation, resulting in therapeutic benefit for inflammation and lung injury triggered by the influenza viruses. In the current study, in vitro and in vivo experiments were carried out to investigate the effects and corresponding underlying mechanisms of RosA on influenza virus-triggered severe pneumonia and lung injury.

## Materials and methods

### Materials

**Fig. 1:**
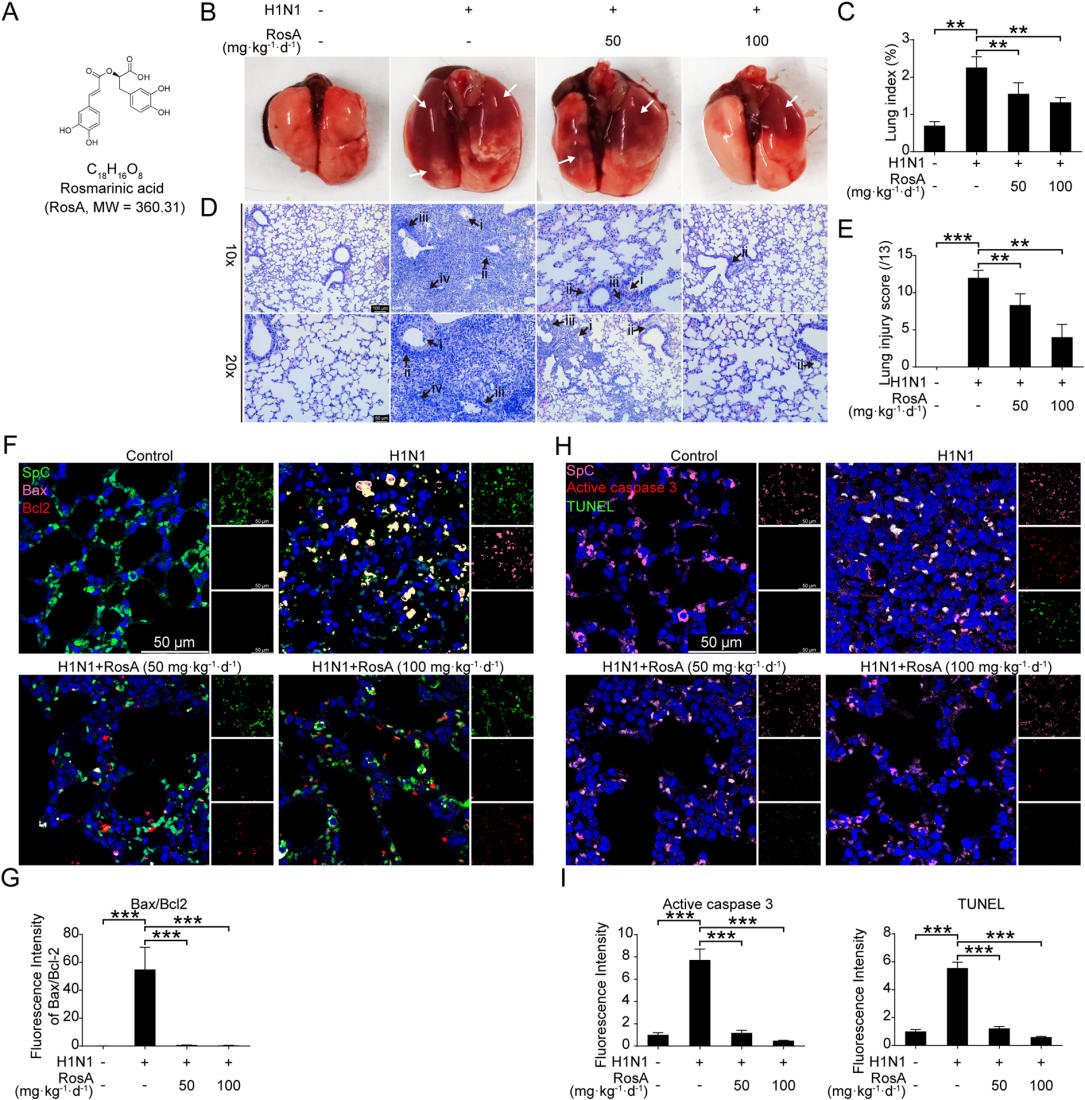
Effects of RosA on H1N1 virus-mediated lung injury in vivo. **A** Chemical structure of RosA. **B** At day 7 p.i., gross examination of the lungs. White arrows indicate the lungs with edema and hemorrhage. **C** Lung index (lung/body weight ratio) was used to assess the severity of exudation and edema. **D** At day 7 p.i., lung histological changes elicited by the H1N1 viruses were examined by H&E staining. Black arrows show: (i) bronchi with epithelial sloughing; (ii) peribronchitis; (iii) perivasculitis; (iv) alveolar collapse and leukocyte infiltration. **E** Histological scoring of lung injury. **F** Representative immunofluorescence images of Bax (pink) and Bcl2 (red) expression in lung SpC^+^ (green) alveolar epithelial cells. **G** Quantitative analysis of fluorescence intensities for Bax and Bcl2 in SpC^+^ alveolar epithelial cells. **H** Representative immunofluorescence images of active caspase 3 (red) and TUNEL assay (green) in lung SpC^+^ (pink) alveolar epithelial cells. **I** Quantitative analysis of fluorescence intensities for active caspase 3 and TUNEL in SpC^+^ alveolar epithelial cells. ^*^P < 0.05, ^**^P < 0.01, ^***^P < 0.001

RosA (Fig. 1A) was purchased from MedChem Express (HY-N0529; Shanghai, China). h-PGDS inhibitor (h-PGDS inhibitor 1; CAS NO. 1033836-12-2) was acquired from AdooQ Bioscience (Irvine, CA, USA). All chemicals were prepared in dimethyl sulfoxide (DMSO) and stored as stock solutions at − 20 ℃. TSAPLus fluorescent triple staining kit was obtained from Servicebio (G1236-100T; Wuhan, China). One-step TUNEL in Situ apoptosis kit (Green, FITC) was obtained from Elabscience Biotechnology Co., Ltd (E-CK-A320; Wuhan, China).

### Viruses

Two strains of influenza viruses, including A/PR/8/34 (H1N1) and A/FM1/H1N1, were provided by Dr. Jing Li from Guangzhou Institute of Respiratory Health & Guangzhou Medical University. The propagation of all virus stains was performed by injection of the diluted viruses in the allantoic cavity of 10-day-old specific pathogen-free embryonated hen’s eggs. Allantoic fluid was harvested for virus titer determination using standard plaque assay in Madin–Darby Canine Kidney (MDCK) cells. Virus stocks were stocked at − 80 ℃ in aliquots.

### Cell culture and treatment

Human alveolar epithelial A549 cells were purchased from ATCC. The cells were cultivated in Dulbecco’s Modified Eagle Medium/Nutrient Mixture F-12 (DMEM/F12, 1:1 mixture) supplemented with 10% fetal bovine serum (FBS) in the humidified incubator (3111; Thermo Fisher Scientific, Waltham, MA, USA) at 37 ℃ in a 5% CO_2_ atmosphere. After reaching confluency, cells were inoculated with indicated A/PR/8/34 (H1N1) viruses diluted in serum-free medium. Then, the virus inoculum was discarded and replaced with serum-free medium containing the indicated concentration of compounds.

### Pro-inflammatory cytokine and PGD_2_ determination

Culture supernatant and lung tissue homogenized in PBS were centrifuged at 13,000*g* for 15 min to remove the cell debris. Levels of pro-inflammatory cytokines in the lung homogenates and culture supernatant were measured using a commercially available multianalyte bead-based kit (Bio-Rad; Hercules, CA, USA) according to the manufacturer’s instructions. PGD_2_ levels in the supernatant were determined by an ELISA kit (E-EL-0066, Elabscience, Wuhan, China).

### Flow cytometry

To measure the effects of RosA on virus-mediated apoptosis, both floating cells in the culture supernatant and adherent cells detached by EDTA-free trypsin were collected. After washing in 1× annexin binding buffer, cells in 100 µL of binding buffer were incubated with Annexin V-FITC and propidium iodide (PI) at room temperature in the dark for 30 min. Then, cells were analyzed by a flow cytometer (LSRII Fortessa; BD, San Jose, CA) within 4 h.

To analyze the levels of CD3^+^CD8^+^ T cells, 20 µL of peripheral blood sample from each group was preincubated with anti-FcγRIII/II (Fc block) (Anti-Mouse CD16/32; 553141). After 30 min of incubation, the following fluorochrome-labelled antibodies: PeCY-7-conjugated CD3 (Clone: 145-2C11; Biolegend), PE-conjugated CD4 (Clone: GK 1.5; Biolegend) and APC-conjugated CD8 (Clone: 53-6.7; Biolegend), were added to the samples for a further 30 min of incubation at room temperature. Afterward, ammonium-chloride-potassium (ACK) lysing buffer was used to lyse red blood cells in the blood samples. Finally, samples were resuspended in 500 µL of staining buffer and analyzed by a flow cytometer (LSRII Fortessa; BD, San Jose, CA).

### Animal experiments

Adult (6- to 8-week-old) female C57BL/6 mice were obtained from Guangdong Medical Laboratory Animal Center (Guangzhou, Guangdong, China) and maintained under germ-free conditions for a week to adapt to the new environment. For RosA administration alone, 32 mice were randomly divided into four groups (n = 8 in each group): (1) the control group; (2) the H1N1 infection group; (3) the RosA treatment group (low dose: 50 mg·kg^−1^·d^−1^); (4) the RosA treatment group (high dose: 100 mg·kg^−1^·d^−1^). For the combination of RosA with h-PGDS inhibitor treatment, 32 mice were randomly divided into four groups as follows: (1) the control group; (2) the H1N1 infection group; (3) the high dose of RosA treatment group (100 mg·kg^−1^·d^−1^); (4) the combination of RosA (100 mg·kg^−1^·d^−1^) with h-PGDS inhibitor (2 mg·kg^−1^·d^−1^) treatment group. Mice were intragastrically administered with the above-mentioned concentrations of RosA for 2 consecutive days before establishment of in vivo viral infection. Mice in the combination of RosA (100 mg·kg^−1^·d^−1^) and h-PGDS inhibitor group were given h-PGDS inhibitor intraperitoneally for 30 min before receiving RosA intragastrically. For viral infection, mice were anesthetized with 10% chloral hydrate (3.5 mL·kg^−1^, i.p.) and inoculated with 5LD_50_ of A/FM1/H1N1 virus (mouse-adapted strain; in 50 µL serum-free DMEM medium) through intranasal route. After that, mice were monitored for another 7 days before being sacrificed.

### Histological analysis

For lung histological examination, mice were anesthetized with 10% chloral hydrate and then the left lobe of the lungs was collected. After fixation in 4% paraformaldehyde for 48 h, lung tissues were dehydrated, transparentized and embedded with increasing concentrations of ethanol (70%, 80%, 95% × 2, 100% × 2), xylene and paraffin, respectively. Afterward, 4-µm-thick lung sections were dewaxed in xylene and rehydrated in a series of descending concentrations of ethanol before staining with H&E. The lung histopathological changes were analyzed under a light microscope (Leica, Wetzlar, Germany). The scores of lung histological changes were evaluated based on airway inflammation, vascular inflammation and parenchymal inflammation, as previously described.

### Immunofluorescence staining

After treatment with or without RosA for 24 h, virus-infected cells were harvested and washed with 1× PBS for three times. Then, cells were permeabilized with 0.5% Triton X-100 for 15 min and blocked with 5% BSA for 30 min at room temperature. Subsequently, cells were incubated with primary antibody against phosphorylated NF‑κB p65 (Ser^536^) (#3033s; Cell Signaling Technology) at 1:200 dilution overnight at 4 ℃. Afterward, cells were incubated with fluorescein isothiocyanate (FITC)-conjugated goat anti-rabbit secondary antibody (Multiscience, Hangzhou, China). 4,6-diaminido-2-phenylindoledihydrochloride (DAPI; C1006) was used for nuclear visualization.

For multi-immunofluorescence staining of lung tissues, the rehydrated lung sections were boiled for 10 min in citrate buffer (10 mM, pH 6.0) for antigen retrieval. After quenching endogenous peroxidase with 3% H_2_O_2_, lung sections were blocked with 10% normal horse serum before being incubated with primary antibodies. After quenching endogenous peroxidase with 3% H_2_O_2_, lung sections were blocked with 10% normal horse serum before being incubated overnight at 4 °C with primary antibodies against SpC (DF6647; Affinity Biosciences), CD8 (DF5126; Affinity Biosciences), IL-6 (DF6087; Affinity Biosciences), TNF-α (AF7014; Affinity Biosciences), IL-8 (DF6998; Affinity Biosciences), MCP-1 (DF7577; Affinity Biosciences), granzyme B (#17215; Cell Signaling Technology), Bax (GTX56246; GeneTex), Bcl2 (GTX100064; GeneTex), and active caspase 3 (GTX22302; GeneTex). Then, horseradish peroxidase (HRP)-conjugated anti-rabbit secondary antibody (#70-GAR007; Multisciences Biotech) was used to probe the specific binding and catalyzed fluorophore-labeled tyramine deposition onto the tissues. To stain another molecule, sections were treated beginning with antigen retrieval to remove binding antibodies, and then repeating the preceding steps until all antigen staining was completed. The stained sections were captured with confocal microscopy (Stellaris; Leica, Wetzlar, Germany).

### Western blot

Total protein was extracted from cultured cells for western blot analysis as previously described. Briefly, cells were lysed in radioimmunoprecipitation assay (RIPA) lysis buffer (Beyotime, Shanghai, China) containing 1% phenylmethanesulfonyl fluoride (PMSF; Beyotime, Shanghai, China) and protease inhibitor cocktail (Sigma, USA). After being centrifuged to remove cellular debris, the supernatant was collected and subjected to quantification of the protein concentration using the Pierce BCA protein assay kit (Thermo Fisher Scientific, Waltham, MA, USA). 25 µg of protein was separated on 10% SDS-PAGE and transferred onto PVDF membranes. After blocking in 5% BSA in 1xTBST (1× TBS, 0.1% (v/v) Tween 20), membranes were incubated overnight at 4 ℃ with the following antibodies, including anti-HO-1 (10701-1-AP, Proteintech), anti-P-IKBα (Ser^32^) (AF1870, Beyotime), anti-IKBα (AF1282, Beyotime), anti-P-p65 (Ser^536^) (3033s, Cell Signaling Technology), anti-p65 (8242s, Cell Signaling Technology), anti-P-ERK1/2 (Thr^202^/Tyr^204^) (4370s, Cell Signaling Technology), anti-ERK1/2 (4695s, Cell Signaling Technology), anti-P-p38 (Thr^180^/Tyr^182^) (4631S, Cell Signaling Technology), anti-P38 (8690s, Cell Signaling Technology), anti-PARP (GTX100573, GeneTex), anti-cleaved caspase 3 (Asp^175^) (9664s, Cell Signaling Technology). Following reacting with HRP-conjugating secondary antibodies, protein bands were visualized with ECL reagents. Using Image J software (version 1.44P), the relative protein expression was quantified by normalizing to GAPDH (AB2000; Abways).

### Plasmid transfection produces

The human h-PGDS-overexpression plasmid (HG15286-ACG) was obtained from Sino Biological. Both the human h-PGDS-overexpression plasmid (0.5 µg) and 5 µL of Lipofectamine 2000 were separately diluted in 250 µL of Opti-MEM medium for 10 min of incubation, and then mixed fully. After another 20 min of incubation, lipoplexes composed of the h-PGDS-overexpression plasmid and Lipofectamine 2000 were added to cells for 6 h. Then, the medium was replaced with fresh, complete medium for cell growth until the confluence reached 90%.

### Statistical analyses

All experimental data are presented as means ± standard deviation (SD). The normality of the data was determined by the Shapiro–Wilk normality test. Data with normal distributions were compared using one-way analysis of variance (ANOVA) followed by Newman–Student–Keuls tests. If the data were not normally distributed, a log_10_ transformation of nonnormally distributed variables yielded a normal distribution and then ANOVA with Newman–Student–Keuls tests was performed on the transformed values. If the log_10_ transformation did not yield a normal distribution, nonparametric tests were performed on the original data. The difference was considered to be statistically significant with *P* value was less than 0.05.

## Results

### RosA prevents H1N1 virus-induced lung damage in vivo

To investigate the protective effects of RosA on H1N1 virus-induced lung injury, mice were intragastrically administrated with RosA for 2 consecutive days prior to viral infection. At day 7 p.i., gross examination of the lungs showed that mice with H1N1 virus infection exhibited severe edema and diffuse hemorrhage in the lung, which were significantly alleviated by RosA administration (Fig. 1B). Moreover, the elevation of lung index (an indicator of lung injury) was reversed by treatment with RosA (Fig. 1C). Meanwhile, the potential effects of RosA on H1N1 virus-elicited lung histopathological changes were evaluated by H&E staining. Mice infected with H1N1 virus alone, when compared to the vehicle and RosA groups, exhibited severe lung pathological changes, including prominent inflammatory cells infiltrated into alveolar space, peritracheal and perivascular tissues, bronchioles filled with lymphocytes, alveolar collapse and sloughed bronchial epithelial cells (Fig. 1D). In contrast, treatment with RosA could remarkably reduce these histopathology changes induced by H1N1 virus. In addition, the lung injury scores of the RosA treatment groups were significantly lower than those of the H1N1 virus infection group (Fig. 1E). Lung tissue apoptosis after H1N1 virus infection has been found to be associated with significantly worsened lung injury. As it is shown in Fig. 1F, viral infection was shown to lower levels of the anti-apoptotic protein Bcl2 (red) and raise levels of the pro-apoptotic protein Bax (pink) in type II alveolar epithelial cells of the lungs (SpC^+^, green) (Fig. 1F and G). In contrast, RosA treatment effectively reversed the H1N1 virus-elicited reduction in Bcl2 expression and enhanced Bax expression (Fig. 1F and G). Furthermore, increased apoptosis of alveolar epithelial cells caused by H1N1 viruses was further confirmed by TUNEL assay (green) and immunofluorescence staining of active caspase 3 (red) (Fig. 1H and I), whereas those were markedly reduced in the lung tissues of mice with RosA administration. Together, these observations suggest that RosA has the capacity to attenuate H1N1 virus-elicited acute lung injury.

### RosA suppresses H1N1 virus-induced excessive inflammation

**Fig. 2:**
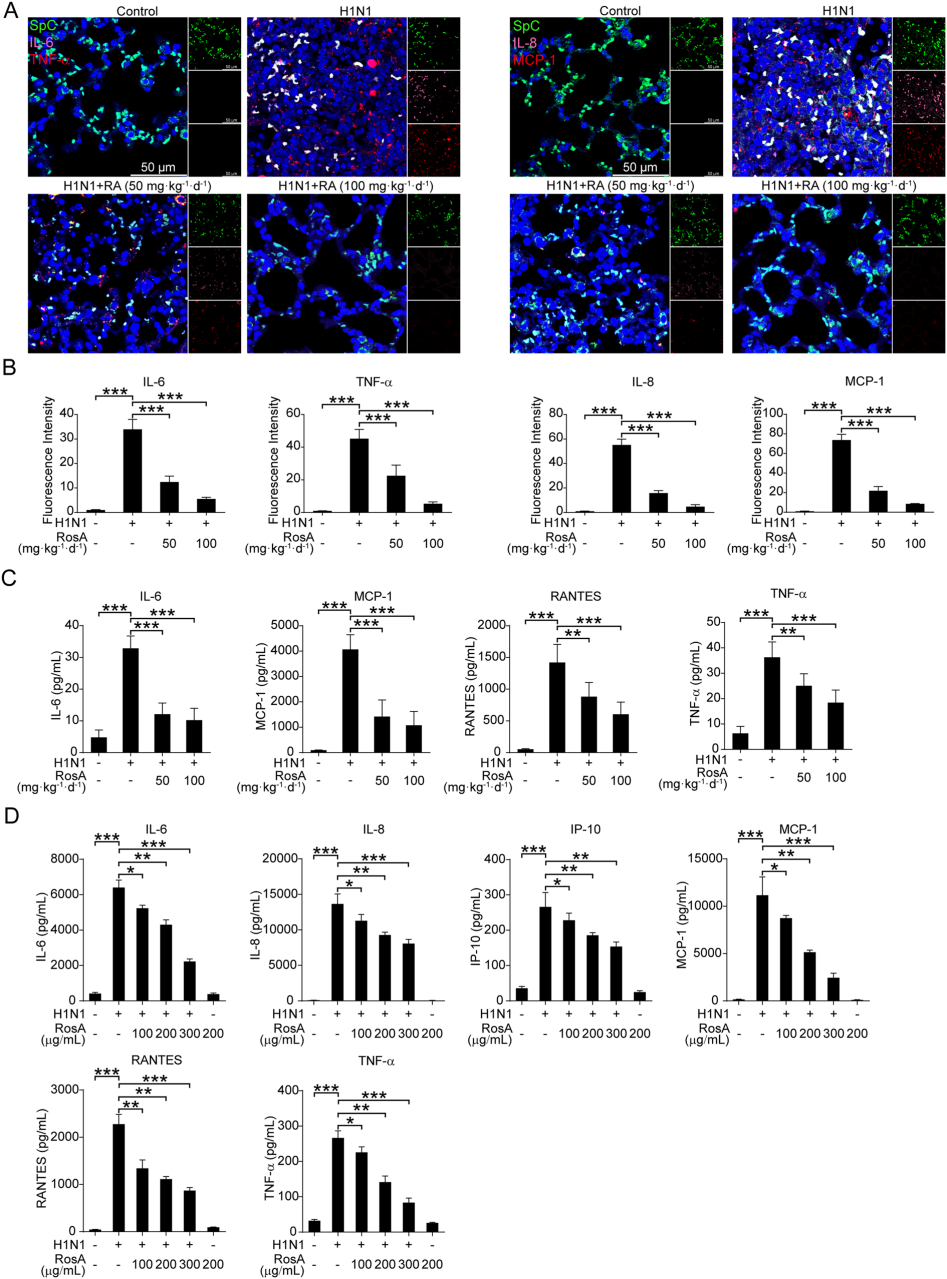
Effects of RosA on the H1N1 virus-triggered pro-inflammatory response. **A** Representative immunofluorescence images of IL-6, IL-8 (pink) and TNF-α, MCP-1 (red) in lung SpC^+^ (green) alveolar epithelial cells. **B** Quantitative analysis of fluorescence intensities for IL-6, TNF-α, IL-8 and MCP-1 in SpC^+^ alveolar epithelial cells. **C** Levels of pro-inflammatory mediators (IL-6, MCP-1, RANTES and TNF-α) in the lung homogenates were determined by Luminex assay. **D** Levels of pro-inflammatory mediators (IL-6, IL-8, IP-10, MCP-1, RANTES and TNF-α) were determined by Luminex assay. ^*^P < 0.05, ^**^P < 0.01, ^***^P < 0.001

The enhanced elevation of pro-inflammatory cytokines in response to virus infection was associated with respiratory illness and clinical symptoms [26]. To reveal whether RosA mitigates H1N1 virus-induced inflammation, we measured the levels of pro-inflammatory cytokines in the lung section by immunofluorescence. The results demonstrated that the expression of IL-6, TNF-α, IL-8 and MCP-1 in lung alveolar epithelial cells (SpC^+^) was remarkably increased in H1N1 virus-infected mice compared with the control group, which was significantly reduced by RosA treatment (Fig. 2A and B). To further confirm the anti-inflammatory property of RosA on H1N1 virus infection, we measured the levels of pro-inflammatory cytokines in the lung homogenates of H1N1 virus-infected mice with or without RosA administration. Similar to the results of pro-inflammatory cytokines in the lung sections, we observed that RosA administration effectively decreased H1N1 virus-elicited increased levels of pro-inflammatory cytokines in the homogenates, including IL-6, MCP-1, RANTES and TNF-α (Fig. 2C). Meanwhile, the effects of RosA on H1N1 virus-mediated pro-inflammatory reactions in vitro were determined by the Luminex assay. As shown in Fig. 2D, the elevation of pro-inflammatory mediators α (IL-6, IL-8, IP-10, MCP-1, RANTES and TNF-α) in H1N1 virus-infected A549 cells was suppressed by RosA treatment in a dose-dependent manner. Therefore, our results demonstrated that RosA has the capacity to suppress H1N1 virus-induced excess inflammation, which might result in protective benefits against H1N1 virus-mediated lung injury.

### RosA inhibits H1N1 virus-mediated activation of NF-κB and P38 MAPK signaling

**Fig. 3:**
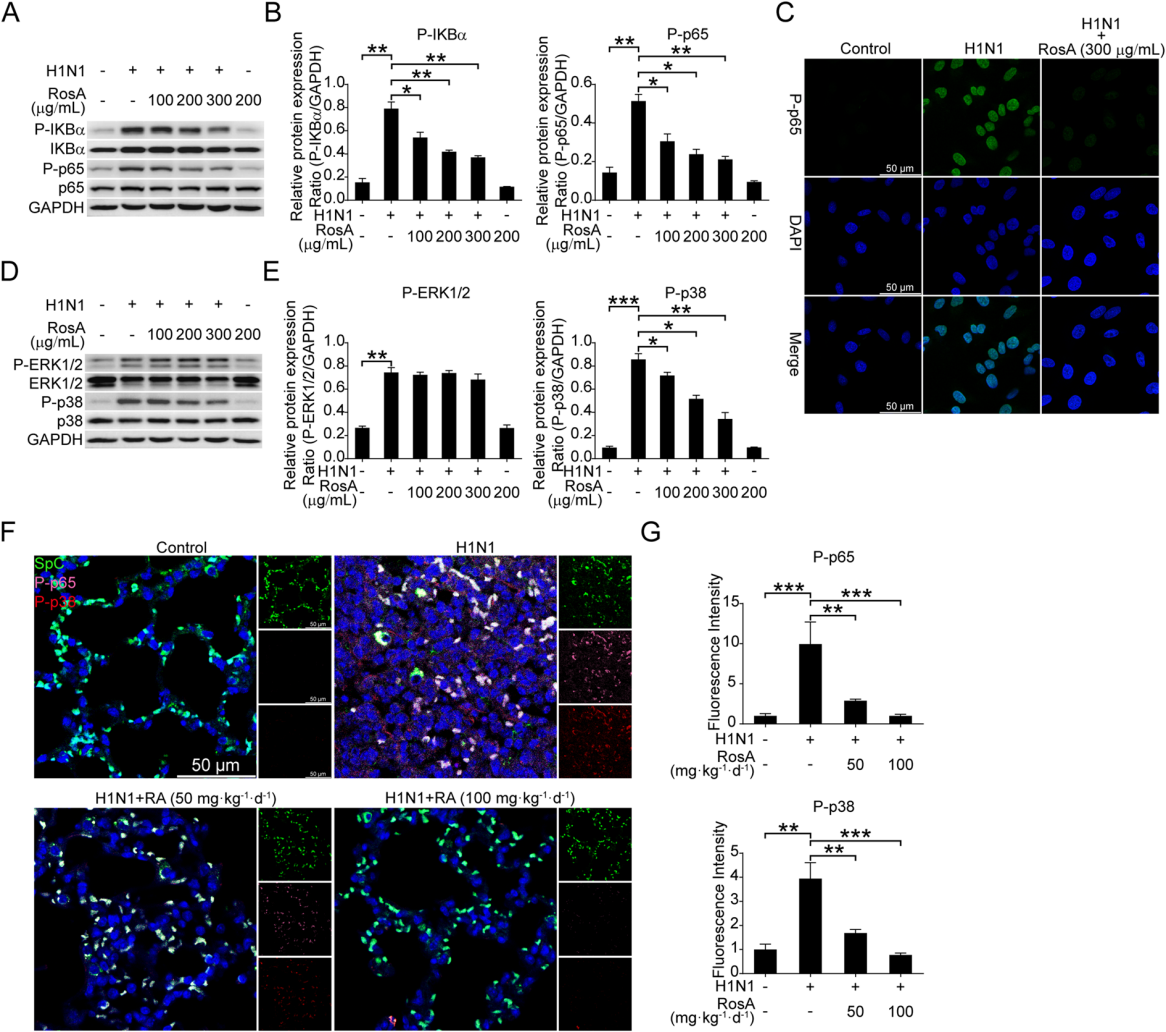
Effects of RosA on H1N1 virus-induced activation of NF-κB and P38 MAPK signaling. **A** Western blotting analysis of NF-κB signaling in H1N1 virus-infected cells. **B** Relative NF-κB signaling-related molecule expression was quantified relative to GAPDH. **C** Immunofluorescence analysis of P-p65 nuclear translocation in H1N1 virus-infected cells. **D** Western blotting analysis of MAPK signaling (ERK1/2 and p38) in H1N1 virus-infected cells. **E** Relative MAPK signaling-related molecule expression was quantified relative to GAPDH. **F** Representative immunofluorescence images of NF-κB (pink) and P-p38 (red) in lung SpC^+^ (green) alveolar epithelial cells. **G** Quantitative analysis of fluorescence intensities for NF-κB and P-p38 in SpC^+^ alveolar epithelial cells. ^*^P < 0.05, ^**^P < 0.01, ^***^P < 0.001

Aberrant activation of NF-κB and P38 MAPK signaling has been demonstrated to be linked to robust cytokine production (also termed “cytokine storm”), resulting in an aggravation of the severity of influenza disease [16, 27]. The above results in Fig. 2A–D indicated that RosA could inhibit H1N1 virus-triggered excessive pro-inflammatory mediator production in vitro and in vivo. Therefore, we sought to investigate whether RosA has the capacity to suppress H1N1 virus-triggered activation of these host signaling pathways. As shown in Fig. 3A and B, H1N1 virus-infected A549 cells with RosA treatment have been found to attenuate the increased activation of NF-κB signaling pathway-related molecules, including P-IKBα and P-p65. Meanwhile, we also found that the nuclear translocation of P-p65 elicited by the H1N1 virus was suppressed by RosA treatment (Fig. 3C). Moreover, RosA treatment was found to suppress H1N1 virus-triggered activation of P-p38 MAPK but did not exhibit inhibitory effects on P-ERK1/2 activation (Fig. 3D and E). Next, we employed multiple-label immunofluorescence experiment to investigate the effects of RosA on H1N1 virus-mediated activation of the NF-κB (pink) and P-p38 MAPK (red) signaling pathways in lung SpC^+^ (green) alveolar epithelial cells. Consistent with the results in vitro, our results showed that the abnormal activation of the NF-κB and P-p38 MAPK signaling pathways in lung SpC^+^ alveolar epithelial cells elicited by viral infection was weakened by RosA administration (Fig. 3F and G). Therefore, these data suggested that RosA suppressed H1N1 virus-triggered excessive inflammation, which was probable due to its inhibitory effects on NF-κB and P38 MAPK signaling.

### Inhibitory effects on H1N1 virus-induced NF-κB and P38 MAPK signaling and excess inflammation by RosA are dependent on increased expression of h-PGDS-PGD_2_-HO-1 signal axis

**Fig. 4:**
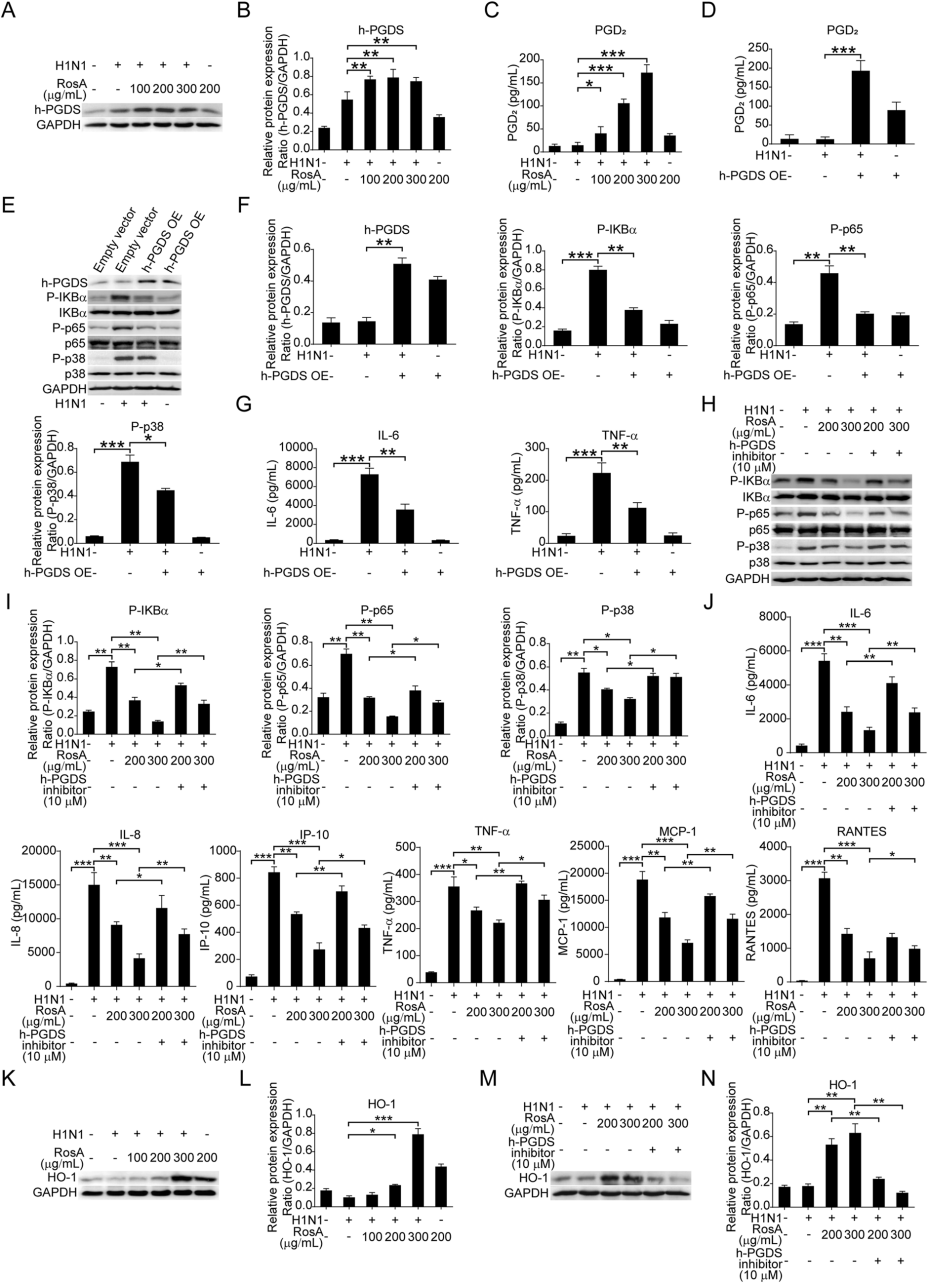

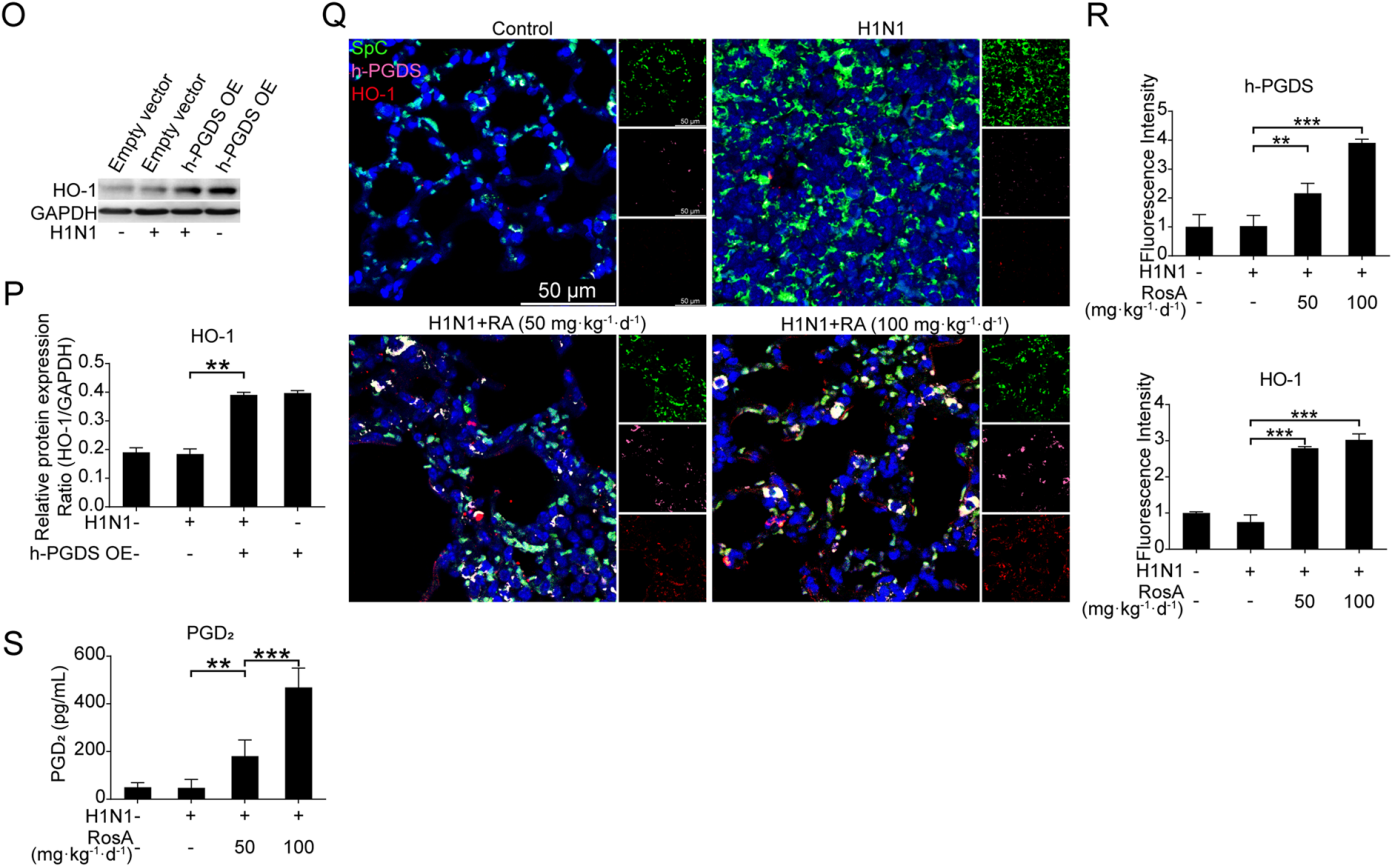
The increased expression of h-PGDS/PGD2/HO-1 was responsible for the inhibitory effects of RosA on H1N1 virus-induced NF-κB and P38 MAPK activation. **A** The expression of h-PGDS in H1N1 virus-infected cells was analyzed by Western blotting. **B** Relative protein expression of h-PGDS was normalized to GAPDH levels. **C** ELISA assay was performed to measure the levels of PGD_2_ in the culture supernatant. **D** The levels of PGD_2_ in the culture supernatant of h-PGDS overexpression (h-PGDS OE) plasmid-transfected A549 cells with or without H1N1 virus infection were quantified by ELISA assay. **E** The levels of P-p65 and P-p38 in h-PGDS overexpression (h-PGDS OE) plasmid-transfected A549 cells with or without H1N1 virus infection were detected by Western blotting. **F** Relative protein expression of h-PGDS, P-IKBα, P-p65 and P-p38 was normalized to GAPDH levels. **G** Luminex assay was performed to measure the levels of pro-inflammatory cytokines (IL-6 and TNF-α) in the culture supernatant of h-PGDS overexpression (h-PGDS OE) plasmid-transfected A549 cells with or without H1N1 virus infection. **H** Western blot analysis of P-IKBα, P-p65 and P-p38 in H1N1 virus-infected cells treated with RosA alone or in combination with h-PGDS inhibitor. **I** The relative expression of P-IKBα, P-p65 and P-p38 expression was quantified relative to GAPDH. **J** Luminex assay were performed to measure the levels of pro-inflammatory cytokines (IL-6, IL-8, IP-10, TNF-α, MCP-1 and RANTES) in H1N1 virus-infected cells treated with RosA alone or in combination with h-PGDS inhibitor. **K** The expression of HO-1 in H1N1 virus-infected cells was detected by Western blotting. **L** HO-1 protein levels were quantified by normalizing to GAPDH levels. **M** Western blot analysis of HO-1 in H1N1 virus-infected cells treated with RosA alone or in combination with h-PGDS inhibitor. **N** Relative HO-1 expression was quantified relative to GAPDH. **O** The levels of HO-1 in h-PGDS overexpression (h-PGDS OE) plasmid-transfected A549 cells with or without H1N1 virus infection were detected by Western blotting. **P** Relative HO-1 expression was quantified relative to GAPDH. **Q** Representative immunofluorescence images of h-PGDS (pink) and HO-1 (red) in lung SpC^+^ (green) alveolar epithelial cells. **R** Quantitative analysis of fluorescence intensities for h-PGDS and HO-1 in SpC^+^ alveolar epithelial cells. **S** The levels of PGD_2_ in the lung homogenates were determined by ELISA assay. ^*^P < 0.05, ^**^P < 0.01, ^***^P < 0.001

Previous studies demonstrated that h-PGDS agonists and the production of h-PGDS-derived PGD_2_ in nonhematopoietic cells (alveolar endothelial and epithelial cells) could be beneficial for alleviating acute lung injury [13]. To reveal whether h-PGDS-PGD_2_ signaling is involved in the protective effects of RosA against H1N1 virus infection, we therefore investigated the expression of h-PGDS in H1N1 virus-infected A549 cells. Interestingly, our results showed that H1N1 virus-infected A549 cells treated with RosA effectively increased expression of h-PGDS (Fig. 4A and B). And the levels of PGD_2_ were elevated by RosA treatment (Fig. 4C). To confirm whether the increased levels of h-PGDS provide protective effects against viral infection, we first examined the levels of PGD_2_ in h-PGDS-overexpressed cells with H1N1 virus infection. As shown in Fig. 4D and F, h-PGDS overexpression plasmid-transfected cells with H1N1 virus infection significantly increased the levels of PGD_2_ in the culture supernatant (Fig. 4D). Next, the effects of h-PGDS overexpression on the activation of virus-elicited host cellular signaling were investigated. We discovered that A549 cells transfected with an h-PGDS overexpression plasmid suppressed H1N1 virus-induced activation of the NF-κB and P38 MAPK signaling pathways (Fig. 4E and F), indicating that h-PGDS may be protective against H1N1 virus infection. And the levels of pro-inflammatory cytokines (IL-6 and TNF-α) were found to be reduced in cells with h-PGDS overexpression (Fig. 4G).

Given that RosA has the capacity to suppress H1N1 virus-induced activation of NF-κB and P38 MAPK signaling, it is interesting to figure out whether the inhibitory effects of RosA on NF-κB and P38 MAPK signaling were attributable to h-PGDS agonists. As expected, our results demonstrated that blockade of h-PGDS by h-PGDS inhibitors abrogated the suppressive effects of RosA on H1N1 virus-elicited NF-κB and P38 MAPK signaling (Fig. 4H and I). And the reduction of the expression of pro-inflammatory mediators (IL-6, IL-8, IP-10, TNF-α, MCP-1, RANTES) by RosA was found to be reversed by treatment with h-PGDS inhibitors (Fig. 4J). Previous studies revealed that h-PGDS-PGD2 signaling transduction has the capacity to up-regulate expression of HO-1, which thus provides a beneficial effect for inflammatory diseases [28, 29]. It is therefore of significance to investigate whether the activation of h-PGDS-PGD_2_ signaling by RosA would lead to an increase in the expression of the HO-1 protein in H1N1 virus-infected A549 cells. As expected, H1N1 virus-infected A549 cells treated with RosA were found to increase the expression of HO-1 (Fig. 4K and L). While, blockade of h-PGDS abrogated the upregulation of HO-1 protein induced by RosA treatment (Fig. 4M and N). Furthermore, cells with h-PGDS overexpression were also found to have elevated the expression of the HO-1 protein (Fig. 4O and P). In addition, results of multiple-label immunofluorescence experiments showed that H1N1 virus-infected mice with RosA administration significantly increased the expression of h-PGDS (pink) and HO-1 (red) in lung SpC^+^ (green) alveolar epithelial cells (Fig. 4Q and R). And the levels of PGD_2_ were increased in the lung homogenates of H1N1 virus-infected mice with RosA treatment (Fig. 4S). Collectively, our results demonstrated that the inhibitory effects of RosA on H1N1 virus-mediated NF-κB and P38 MAPK signaling and excess inflammation were attributed to activation of h-PGDS-PGD_2_-HO-1 signaling.

### Involvement of h-PGDS in the suppression of H1N1 virus-induced apoptosis by RosA

**Fig. 5:**
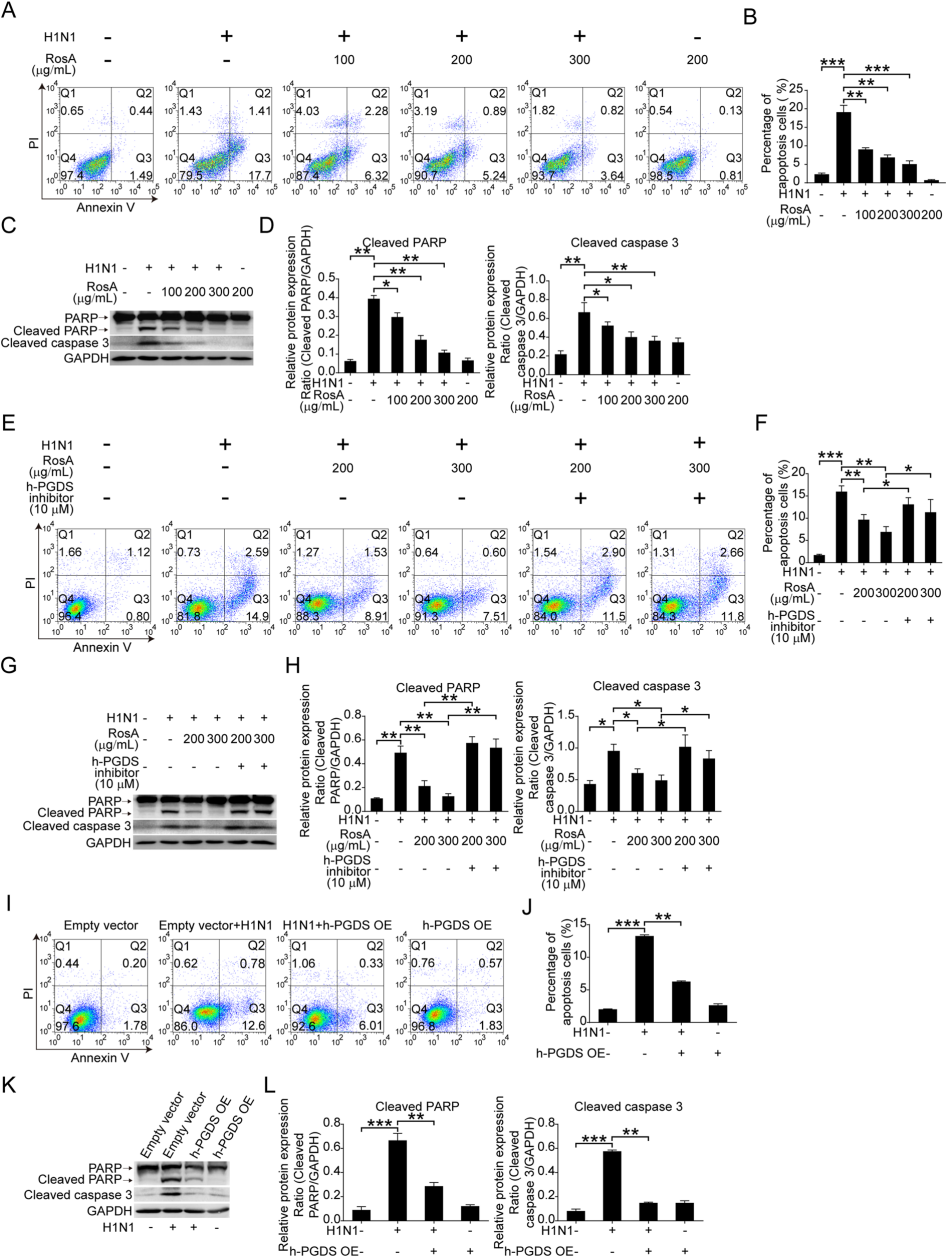

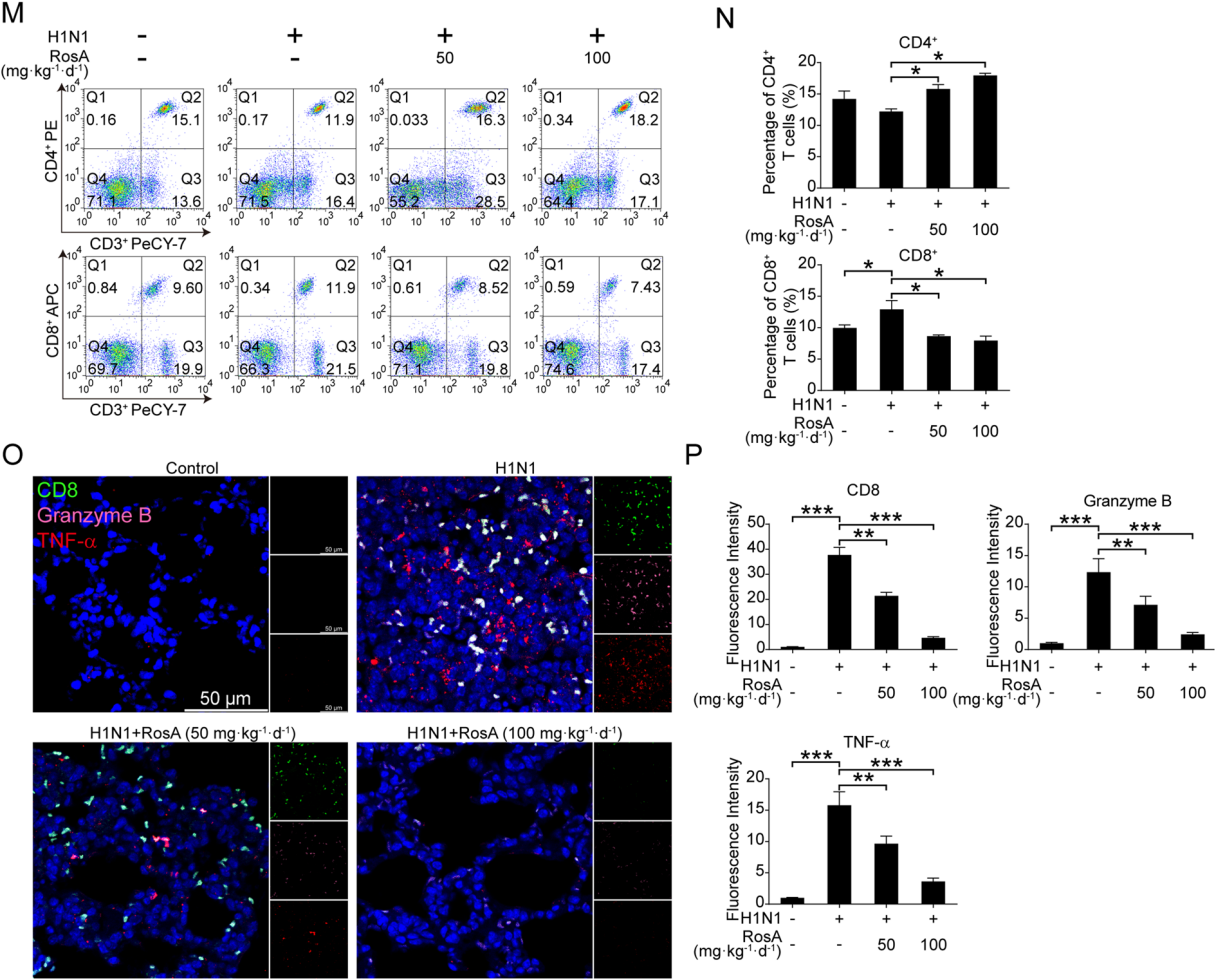
Effects of RosA on H1N1 virus-induced apoptosis. **A** The apoptosis of H1N1 virus-infected cells was harvested for flow cytometry analysis after RosA treatment for 24 h. **B** Apoptosis percentage in H1N1 virus-infected A549 cells treated with or without RosA. **C** Western blot analysis of cleaved PARP and cleaved caspase 3 in H1N1 virus-infected cells treated with RosA. **D** The relative expression of cleaved PARP and cleaved caspase 3 was quantified relative to GAPDH. **E** H1N1 virus-infected A549 cells were treated with RosA alone or in combination with h-PGDS inhibitor for 24 h. Flow cytometry was used to identify the apoptosis of these cells. **F** The percentage of apoptosis in A549 cells with H1N1 virus-infected A549 cells treated for 24 h with RosA alone or in conjunction with an h-PGDS inhibitor. **G** Western blot analysis of cleaved PARP and cleaved caspase 3 in H1N1 virus-infected cells treated with RA alone or in combination with h-PGDS inhibitor. **H** The relative expression of cleaved PARP and cleaved caspase 3 expression was quantified relative to GAPDH. **I** The apoptosis of cells in h-PGDS overexpression (h-PGDS OE) plasmid-transfected A549 cells with or without H1N1 virus-infection were detected by flow cytometry. **J** The percentage of apoptosis in h-PGDS overexpression (h-PGDS OE) plasmid-transfected A549 cells with or without H1N1 virus infection. **K** The levels of cleaved PARP and cleaved caspase 3 in h-PGDS overexpression (h-PGDS OE) plasmid-transfected A549 cells with or without H1N1 virus infection were detected by Western blotting. **L** The relative expression of cleaved PARP and cleaved caspase 3 expression was quantified relative to GAPDH. **M** Analysis of CD3^+^CD8^+^ T lymphocytes by flow cytometry from peripheral blood. **N** Quantification of the proportions of CD3^+^CD8^+^ T lymphocytes in the peripheral blood of H1N1 virus-infected mice. **O** Immunofluorescence staining of Granzyme B (pink) and TNF-α (red) in CD8^+^ T lymphocytes (green) of the lung tissues. **P** The relative fluorescence intensities of Granzyme B and TNF-α in CD8^+^ T lymphocytes were calculated. ^*^P < 0.05, ^**^P < 0.01, ^***^P < 0.001

Alveolar epithelial cell apoptosis has been found to be linked to the detrimental outcome of influenza diseases [30]. The above results in Fig. 1E–I demonstrated that mice with RosA administration could alleviate H1N1 virus-mediated apoptosis of type II alveolar epithelial cells in vivo. We thus investigated whether the activation of h-PGDS-PGD_2_-HO-1 signaling by RosA was related to its anti-apoptotic effects against H1N1 virus-triggered injury in vitro. First, our results demonstrated that the increased apoptosis of H1N1 virus-infected A549 cells was effectively suppressed by RosA treatment (Fig. 5A and B). And we confirmed the anti-apoptotic effects of RosA by detecting H1N1 virus-elevated expression of cleaved PARP and cleaved caspase 3, which were decreased by RosA treatment (Fig. 5C and D). However, H1N1 virus-infected A549 cells with the combination of RosA and h-PGDS inhibitor treatment have been found to weaken the inhibitory effects of RosA on H1N1 virus-elicited apoptosis (Fig. 5E and F). And the reduced expression of apoptosis biomarkers cleaved PARP and cleaved caspase 3 was also reversed by treatment with a combination of RosA and h-PGDS inhibitor (Fig. 5G and H). Moreover, we also demonstrated that A549 cells with h-PGDS overexpression could reduce apoptosis (Fig. 5I and J) and the expression of cleaved PARP and cleaved caspase 3 (Fig. 5K and L) triggered by H1N1 viruses, indicating that h-PGDS has anti-apoptosis properties. Secretion of granzyme B and TNF-α by cytotoxic CD8^+^ T cells could result in nonspecific lysis of uninfected lung epithelial cells, leading to aggravated H1N1 virus-mediated lung injury [31]. Given that RosA alleviated H1N1 virus-mediated lung injury, we thus investigated whether RosA treatment affected the increased levels of cytotoxic CD8^+^ T cells and the production of effectors by CD8^+^ T cells. Flow cytometry detection of cytotoxic CD8^+^ T cells in peripheral blood showed that the levels of cytotoxic CD8^+^ T cells were increased in response to H1N1 virus infection, which was found to be decreased by RosA treatment (Fig. 5M and N). Meanwhile, compared to the H1N1 virus infection group, we also found that H1N1 virus-infected mice with RosA treatment had lower levels of CD8^+^ (green) T cells and the expression of cytotoxic effectors Granzyme B (pink) and TNF-α (red) in the lung tissues (Fig. 5O and P). Therefore, these results suggested that activation of h-PGDS by RosA led to suppression of H1N1 virus-induced apoptosis of alveolar epithelial cells and cytotoxic CD8^+^ T lung recruitment, facilitating amelioration of H1N1 virus-mediated lung injury.

### Inhibition of h-PGDS reverses the protected effects of RosA on H1N1 virus-mediated lung injury

**Fig. 6:**
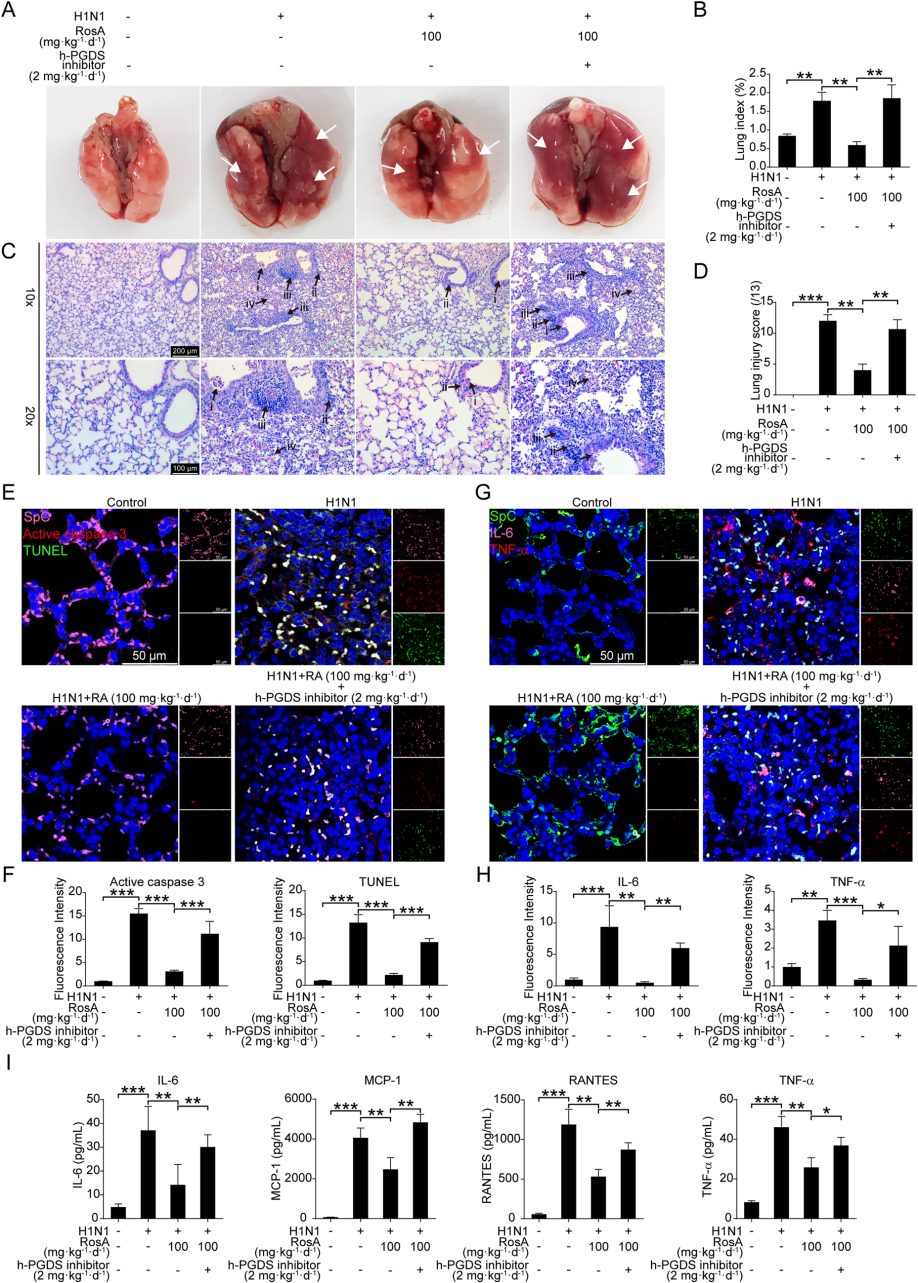
Effects of h-PGDS inhibition on RosA’s protective effects against H1N1 virus-mediated lung injury. Two days before infection with mouse-adapted H1N1 virus (5LD_50_), mice in the group with the combination of RosA with h-PGDS inhibitor were treated intraperitoneally with h-PGDS inhibitor for 30 min, and then administrated intragastrically (i.g.) with RosA. **A** At day 7 p.i., gross pathology showed edema and hemorrhage (white arrows) in the lung tissues. **B** The severity of exudation and edema were elevated by the lung index (lung/body weight ratio). **C** At day 7 p.i., H&E staining was used to assess the lung histological alterations caused by H1N1 viruses. Black arrows show: (i) bronchi with epithelial sloughing; (ii) peribronchitis; (iii) perivasculitis; (vi) alveolar collapse and leukocyte infiltration. **D** Histological scoring of lung injury. **E** Representative immunofluorescence images of active caspase 3 (red) and TUNEL (green) assay in lung SpC^+^ (pink) alveolar epithelial cells. **F** Quantitative analysis of fluorescence intensities for active caspase 3 and TUNEL in SpC^+^ alveolar epithelial cells. **G** The expression of IL-6 and TNF-α in lung SpC^+^ alveolar epithelial cells was detected by immunofluorescence. **H** Quantitative analysis of fluorescence intensities for IL-6 (pink) and TNF-α (red) in SpC^+^ (green) alveolar epithelial cells. **I** Levels of pro-inflammatory mediators (IL-6, MCP-1, RANTES and TNF-α) in the lung homogenates were determined by Luminex assay. ^*^P < 0.05, ^**^P < 0.01, ^***^P < 0.001

To confirm that RosA protected against H1N1 virus-elicited lung injury via activation of h-PGDS signaling, H1N1 virus-infected mice were intraperitoneally injected with h-PGDS inhibitors before RosA administration. We found that treating mice with RosA obviously ameliorated the gross anatomic pathology changes, including lung hemorrhage and edema, which were abolished when combined with h-PGDS inhibitors (Fig. 6A). Meanwhile, the lower levels of lung index were reversed in RosA-administrated mice with the combination of h-PGDS inhibitor treatment (Fig. 6B). Additionally, blockage of h-PGDS decreased the beneficial effects of RosA on H1N1 virus-induced lung pathological alterations, such as alveolar collapse, leukocyte lung parenchyma infiltration, bronchiolitis, and vasculitis (Fig. 6C). Accordingly, the reduction in lung histological scores that resulted from RosA administration was reversed in mice with h-PGDS inhibition (Fig. 6D). Furthermore, results of four-color immunofluorescence staining showed that inhibition of h-PGDS abrogated the inhibitory effect of RosA on the positive signals for TUNEL (green) and active caspase 3 (red) in lung type II alveolar epithelial cells (SpC^+^, pink) (Fig. 6E and F). Meanwhile, a similar trend was observed in the expression levels of pro-inflammatory mediators (IL-6, TNF-α, MCP-1 and RANTES) in lung type II alveolar epithelial cells (SpC^+^) (Fig. 6G and H) as well as lung homogenates (Fig. 6I). Therefore, these results demonstrated that the inhibitory effects of RosA on H1N1 virus-induced apoptosis and inflammation in lung alveolar epithelial cells were dependent on its h-PGDS-activated property.

## Discussion

Excessive pro-inflammatory mediator production and aberrant apoptosis of alveolar epithelial cells are the critical contributors that lead to critically ill patients with influenza infection [26]. Few effective therapeutic options appear to be available for reducing inflammation and apoptosis caused by the influenza virus. Although corticosteroids are commonly used to reduce influenza-related inflammation and acute lung injury, their therapeutic efficacy in patients with severe influenza pneumonia remains controversial [8]. And patients with influenza-associated ARDS treated with corticosteroids have been reported to have not only iatrogenic adverse effects but even increased mortality [32, 33]. Therefore, novel therapeutic approaches for influenza illness treatment should continue to be developed. In the present study, we investigated the effects of RosA on H1N1 virus-mediated inflammation and lung injury in vitro and in vivo. Our results demonstrated that RosA treatment could protect against lethal doses of H1N1 virus-induced severe pneumonia and lung injury in vivo. In vitro experiments showed that A549 cells with overexpression of h-PGDS were found to decrease H1N1 virus-induced inflammation and apoptosis. Interestingly, we further revealed the mechanism by which RosA attenuates H1N1 virus-mediated excessive inflammation and apoptosis linked to activation of the h-PGDS-PGD_2_-HO-1 signaling axis, resulting in suppression of NF-κB and P-p38 MAPK activation. Moreover, we confirmed that blockade of h-PGDS abolished the protective effects of RosA against H1N1 virus-elicited lung injury in vivo.

PGD_2_ synthases are classified into two isoforms that is lipocalin and hematopoietic type [34]. Lipocalin-type PGD synthase (L-PGDS) is found predominantly in the central human nervous system, retina, testis, vasculature, and heart, whereas the distribution of h-PGDS is distinct from that of L-PGDS, which is generally expressed in immune cells [34]. The generation of PGD_2_ from its precursor PGH_2_ is catalyzed by the action of both L-PGDS and h-PGDS [34]. A growing body of research has shown convincingly that two forms of PGD2 synthases (L-PGDS and h-PGDS) and their derived PGD_2_ have immunomodulatory effects against acute or chronic inflammatory diseases [35]. For instance, a previous study showed that lacking of L-PGDS contributed to the development of age-related osteoarthritis [36]. Dextran sulfate sodium (DSS)-induced chronic experimental colitis has been discovered to be exacerbated in mice with the genetic deficiency of h-PGDS [11]. As key therapeutic effectors, elevated expression of h-PGDS and PGD_2_ in adipose mesenchymal stem cells has been reported to decrease expression of TNF-α, IL-6, IL-1β and iNOS in experimental diabetic wounds [37]. In the scenario of acute inflammatory disorder, the roles of L-PGDS and h-PGDS have been shown to be beneficial for attenuating hydrochloride (HCl) or LPS-mediated acute lung injury, respectively [13, 38]. The findings from these series of studies supported the idea that the loss of protective effects in mice with genetic deficiency of L-PGDS or h-PGDS is at least partially due to a lack of PGD_2_ synthesis. However, the pathophysiological role of L-PGDS, h-PGDS or their derived PGD_2_ in response to influenza virus infection remains largely unknown. Given that inflammation disorders and acute lung injury lead to a worse outcome of influenza illness, we thus hypothesized that activation of h-PGDS/PGD_2_ may protect against severe influenza diseases. In order to effectively attenuate influenza-associated illness via an h-PGDS agonist, our study further identified that RosA treatment was found to significantly increase expression of h-PGDS and its downstream product PGD_2_ in vitro and in vivo. Therefore, RosA is an attractive candidate for h-PGDS agonist to relieve the severity of influenza diseases. Indeed, results from in vivo experiments demonstrated that RosA treatment effectively reduced H1N1 virus-mediated lung injury, while blockade of h-PGDS was unexpected to completely reverse these protective effects of RosA, supporting the idea that h-PGDS activation could act as a suitable target for influenza illness.

The multiple biological activities of RosA (e.g., anticancer, cardioprotection and neuroprotection) were reported to be related to its wide range of targets, such as suppression of STAT3 and NF-κB, as well as activation of Nrf2, PPAR-γ [24, 39, 40]. However, RosA with h-PGDS-PGD_2_-activated properties to exert its protective effects against virus-mediated lung injury has not been reported before. During lethal influenza virus infection, aberrant activation of NF-κB and P38 MAPK signaling contributes to excess pro-inflammatory mediator production, which is found to be closely related to the severity of influenza virus-mediated lung injury [16, 27, 41]. Accumulated studies have demonstrated that RosA possesses immunomodulatory effects for alleviating chronic or acute inflammation diseases (such as arthritis, colitis, asthma and lung injury) through acting on multiple molecular targets, including NF-kB, p-STAT3, and MAPKs [23]. Consistent with these findings, our results showed that RosA treatment prominently suppressed H1N1 virus-elicited activation of NF-κB and P38 MAPK signaling, accompanied by a reduction of a series of pro-inflammatory cytokines and chemokines (IL-6, IL-8, IP-10, MCP-1, RANTES and TNF-α). Moreover, h-PGDS inhibition was found to diminish the inhibitory effects of RosA on H1N1 virus-triggered activation of NF-κB and P38 MAPK signaling, as well as these pro-inflammatory mediators. Although the anti-inflammatory effects of RosA have been reported in other studies, it is the first time in our study to reveal that the suppression of H1N1 virus-associated excessive inflammation by RosA treatment has been linked to increased levels of h-PGDS, which thus leads to inhibition of NF-κB and P38 MAPK signaling. Previous studies have reported that the immunomodulatory effects of the h-PGDS-PGD_2_ signal axis are due to the inactivate of NF-κB and P38 MAPK pathway. In a cerebral ischemia injury model, h-PGDS has been found to have the capacity to inactivate of NF-κB signaling, hence exerting anti-inflammatory properties [42]. And the signaling transduction of PGD_2_ through the DP1 receptor in bacteria-mediated endometritis in dairy cows was found to suppress activation of NF-κB and P38 MAPK signaling, thus reducing expression of IL-6, IL-1β, and TNF-α [12]. With regard to the effects of h-PGDS on H1N1 virus-mediated signaling, h-PGDS overexpression was found to reduce H1N1 virus-mediated activation of NF-κB and P38 MAPK. Meanwhile, our results showed that the increased production of pro-inflammatory mediators (IL-6 and TNF-α) triggered by H1N1 virus infection was significantly decreased in A549 cells with h-PGDS overexpression. Our study revealed the mechanism, for the first time, by which h-PGDS has the capacity to decrease H1N1 virus-induced activation of NF-κB and P-p38 signaling, and subsequently attenuate aberrant pro-inflammatory reactions. Furthermore, PGD_2_ has been shown to increase the expression of HO-1, an enzyme that catalyzes the degradation of heme to ferrous iron (Fe^2+^), carbon monoxide (CO), and biliverdin/bilirubin. 15d-PGJ_2_, a metabolite of PGD_2,_ was also reported to be involved in promoting the expression of HO-1 for alleviating influenza pneumonia [43]. The antioxidative and anti-inflammatory functions of HO-1 and its end products could exert a protective role against infectious diseases, including influenza illness [44, 45]. Accordingly, our results showed that h-PGDS overexpression effectively increased the expression of HO-1 in H1N1 virus-infected cells. Previous studies have shown that the therapeutic efficacy of RosA treatment on various stimulus-mediated lung injuries was due to increased levels of HO-1 [46, 47], but it is unclear whether the upregulation of HO-1 by RosA is associated with its elevated effects on h-PGDS during virus infection. Similar to other studies, we also found that H1N1 virus-infected cells with RosA treatment significantly upregulated the levels of HO-1. In order to clarify the relationship between the elevation of HO-1 and h-PGDS in RosA-treated cells, inhibition of h-PGDS was found to abrogate the increased expression of HO-1 induced by RosA, suggesting the upregulation of HO-1 by RosA was dependent on h-PGDS. Therefore, our findings add to the aforementioned studies that the involvement of the h-PGDS-PGD_2_-HO-1 signaling axis contributes to the inhibitory effects of RosA on H1N1 virus-induced aberrant activation of NF-κB and P38 MAPK, thus leading to a reduction of excess inflammation reactions.

Moreover, the aberrant alveolar epithelial cell apoptosis elicited by influenza virus infection can contribute to the destruction of respiratory units, leading to severe lung injury and fatal hypoxemia in patients with influenza virus infection [48]. In fact, the production of PGD_2_ by the catalyzed action of L-PGDS or h-PGDS has been found to prevent hypoxic-ischemic brain injury through inhibition of endothelial cell apoptosis [49]. In an LPS-induced acute lung injury model, PGD_2_ has been shown to suppress LPS-induced apoptosis in vitro and in vivo and hence lessen lung injury [50]. Our results showed that cells with h-PGDS overexpression significantly reduced H1N1 virus-mediated apoptosis. Since increased levels of PGD_2_ in h-PGDS overexpression cells were observed, we therefore supposed that activation of the h-PGDS-PGD_2_ signal possessed anti-apoptosis capabilities in H1N1 virus-infected cells. A previous report showed that RosA has the capacity to reduce the apoptosis of alveolar epithelial cells during ischemia-reperfusion-induced lung injury [46]. Likewise, we found that RosA treatment effectively decreased H1N1 virus-triggered apoptosis of alveolar epithelial cells in vitro and in vivo. Moreover, we also found that the inhibitory effects of RosA on H1N1 virus-elicited apoptosis were abolished by h-PGDS inhibition, suggesting that the anti-apoptotic effects of RosA were dependent on h-PGDS activation. Furthermore, h-PGDS overexpression increased the expression of HO-1 and RosA upregulated HO-1 expression in a h-PGDS-dependent manner. HO-1 and its downstream products (Fe^2+^, CO, and biliverdin/bilirubin) could suppress apoptosis triggered by various stimulations or viruses [51, 52]. Therefore, the apoptotic effects of RosA might be associated with h-PGDS-dependent HO-1 upregulation. Besides, the activation of NF-κB and P38 MAPK pathways elicited by the H1N1 virus was involved in the apoptotic process through the elevation of pro-apoptotic factors (Fas L and TRAIL) [53]. The inhibitory effects of RosA on H1N1 virus-mediated activation of the NF-κB and P38 MAPK pathways as well as apoptosis were reversed when h-PGDS was suppressed. Therefore, we supposed that the protective effects of RosA against H1N1 virus-elicited apoptosis were due to the activation of the h-PGDS-PGD_2_-HO-1 signal axis, leading to inhibition of the NF-κB and P38 MAPK pathways and thus exerting anti-apoptosis effects.

## Conclusions

**Fig. 7:**
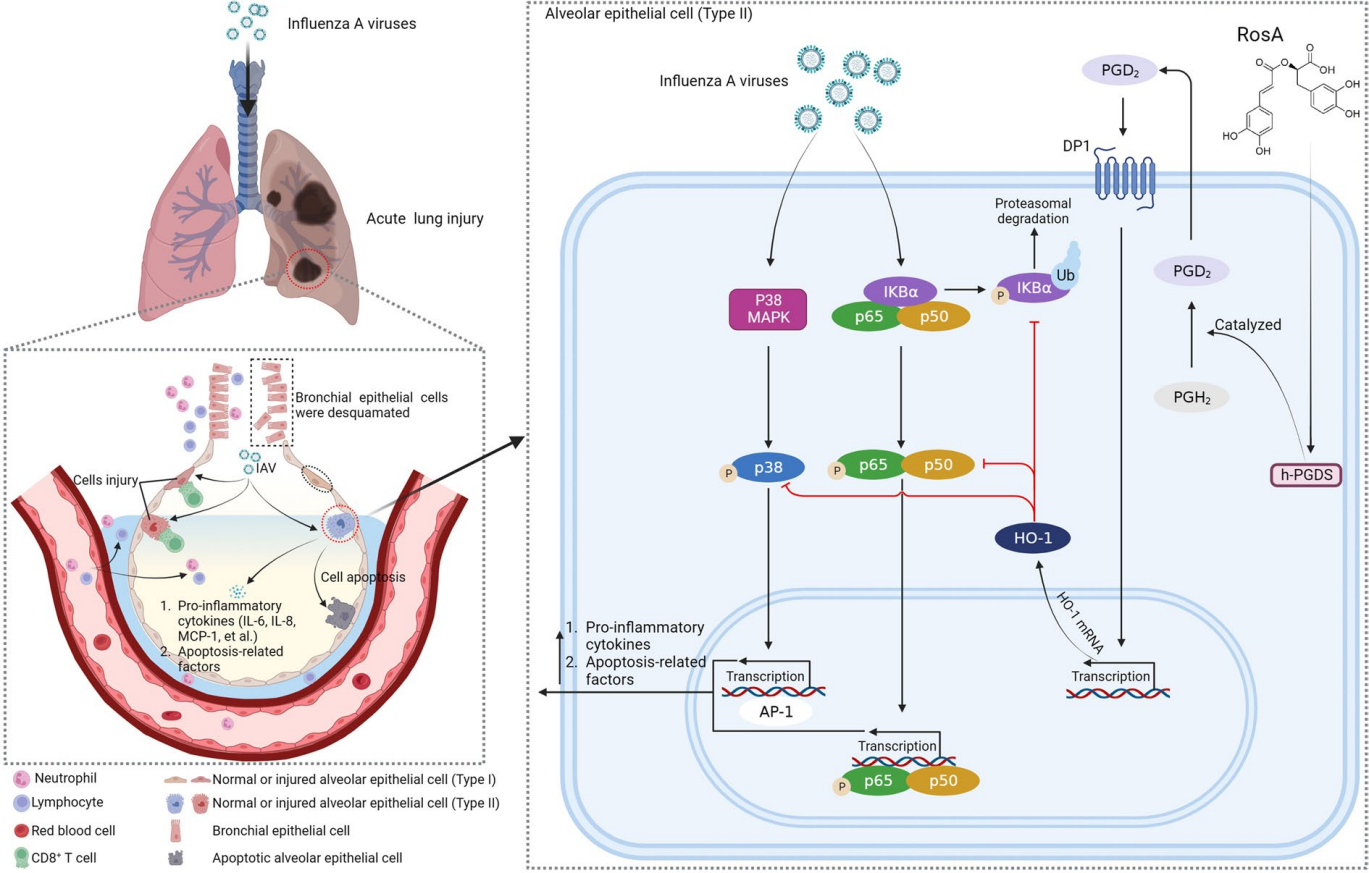
Schematic diagram depicting the mechanism by which RosA prevents H1N1 virus-induced severe lung injury. RosA treatment effectively increases the expression of h-PGDS, which catalyzes the conversion of PGH_2_ to PGD_2_. Subsequently, PGD_2_ binds to its receptor DP1, and thus triggers the expression of HO-1. The increased expression of HO-1 leads to attenuation of H1N1 virus-activated NF-κB and P38 MAPK signaling pathways, resulting in suppression of H1N1 virus-elicited pro-inflammatory responses and apoptosis

Based on these findings, we conclude that the activation of the h-PGDS-PGD_2_-HO-1 signal axis by RosA exerted inhibitory effects on H1N1 virus-mediated NF-κB and P38 MAPK pathway activation, which resulted in a reduction of excess pro-inflammatory response and apoptosis (Fig. 7). Therefore, RosA is an attractive candidate for the development of a new agent to relieve the severity of influenza diseases.

## Data Availability

The data used to support the findings of this study are available from the corresponding author upon request.

## Abbreviations

BSA: Bovine serum albumin
DAPI: 4ʹ,6-Diamidino-2-phenylindole
DMEM: Dulbecco’s modified Eagles medium
DMSO: Dimethyl sulfoxide
ECL: Enhanced chemiluminescence
FITC: Fluorescein isothiocyanate
GAPDH: Glyceraldehyde 3-phosphate dehydrogenase
HRP: Horseradish peroxidase
h-PGDS: Hematopoietic-type PGD_2_ synthase
H&E: Hematoxylin and eosin
MTT: 3-(4,5-dimethylthiazol-2-yl)-2,5-diphenylte-trazolium bromide
PGH_2_: Prostaglandin H2
PGD_2_: Prostaglandin D2
PI: Propidium iodide
PVDF: Polyvinylidene fluoride
RIPA: Radio-immunoprecipitation
